# Enzyme controlled glucose auto-delivery for high cell density cultivations in microplates and shake flasks

**DOI:** 10.1186/1475-2859-7-31

**Published:** 2008-11-18

**Authors:** Johanna Panula-Perälä, Juozas Šiurkus, Antti Vasala, Robert Wilmanowski, Marco G Casteleijn, Peter Neubauer

**Affiliations:** 1 Bioprocess Engineering Laboratory, Department of Process and Environmental Engineering and Biocenter Oulu, University of Oulu, P.O. Box 4300, FI-90014 Oulu, Finland; 2 Fermentas UAB, V. Graiciuno 8, LT-02241 Vilnius, Lithuania; 3 BioSilta Oy, P. O. Box 4300, FI-90014 Oulu, Finland; 4 Department of Biotechnology, Technische Universität Berlin, Ackerstr. 71-76, D-13355 Berlin, Germany

## Abstract

**Background:**

Here we describe a novel cultivation method, called EnBase™, or enzyme-based-substrate-delivery, for the growth of microorganisms in millilitre and sub-millilitre scale which yields 5 to 20 times higher cell densities compared to standard methods. The novel method can be directly applied in microwell plates and shake flasks without any requirements for additional sensors or liquid supply systems. EnBase is therefore readily applicable for many high throughput applications, such as DNA production for genome sequencing, optimisation of protein expression, production of proteins for structural genomics, bioprocess development, and screening of enzyme and metagenomic libraries.

**Results:**

High cell densities with EnBase are obtained by applying the concept of glucose-limited fed-batch cultivation which is commonly used in industrial processes. The major difference of the novel method is that no external glucose feed is required, but glucose is released into the growth medium by enzymatic degradation of starch. To cope with the high levels of starch necessary for high cell density cultivation, starch is supplied to the growing culture suspension by continuous diffusion from a storage gel.

Our results show that the controlled enzyme-based supply of glucose allows a glucose-limited growth to high cell densities of OD_600 _= 20 to 30 (corresponding to 6 to 9 g l^-1 ^cell dry weight) without the external feed of additional compounds in shake flasks and 96-well plates. The final cell density can be further increased by addition of extra nitrogen during the cultivation. Production of a heterologous triosphosphate isomerase in *E. coli *BL21(DE3) resulted in 10 times higher volumetric product yield and a higher ratio of soluble to insoluble product when compared to the conventional production method.

**Conclusion:**

The novel EnBase method is robust and simple-to-apply for high cell density cultivation in shake flasks and microwell plates. The potential of the system is that the microbial growth rate and oxygen consumption can be simply controlled by the amount (and principally also by the activity) of the starch-degrading enzyme. This solves the problems of uncontrolled growth, oxygen limitation, and severe pH drop in shaken cultures. In parallel the method provides the basis for enhanced cell densities. The feasibility of the new method has been shown for 96-well plates and shake flasks and we believe that it can easily be adapted to different microwell and deepwell plate formats and shake flasks. Therefore EnBase will be a helpful tool especially in high throughput applications.

## Background

The fast development in the molecular biodisciplines, including large-scale genome sequencing [1,2], metagenomic approaches [3,4], screening of protein libraries [5], high throughput proteomics [6,7], high throughput protein crystallisation [8-10], and new vector libraries for fast bioprocess development [11,12] set an urgent demand for reliable, efficient, and high-throughput cell cultivation.

Cell cultivation today in laboratory scale is mainly based on shaken cultures which are generally performed as batch cultures (i.e. all nutrients are added at the start of the cultivation). Compared to industrial fed-batch processes, these shaken cultures are characterised by low volumetric cell and product yields. They usually display big culture to culture variation caused by oxygen limitation, overflow metabolism, and pH drop [13-15]. With a further reduction in culture volume, for instance by cultivation in 96 or 384 well plates, variation can be highly impacted by evaporation or condensation in the enclosed wells.

Shaken cultures provide only limited information for bioprocess development; cultivation parameters are often re-optimised in the fermentation laboratories and often the whole process strategy is changed. This significantly increases time and labour costs [13,14]. The problems of variation and scalability of shake flask cultures are generally known, but there is no simple solution. To avoid scale complications a large international consortium of scientists (SPINE-2, ) recently recommended to skip small-scale optimisation tests before the final shake flask scale production of recombinant proteins if this is feasible for the number of constructs to be tested [16].

**Figure 1:**
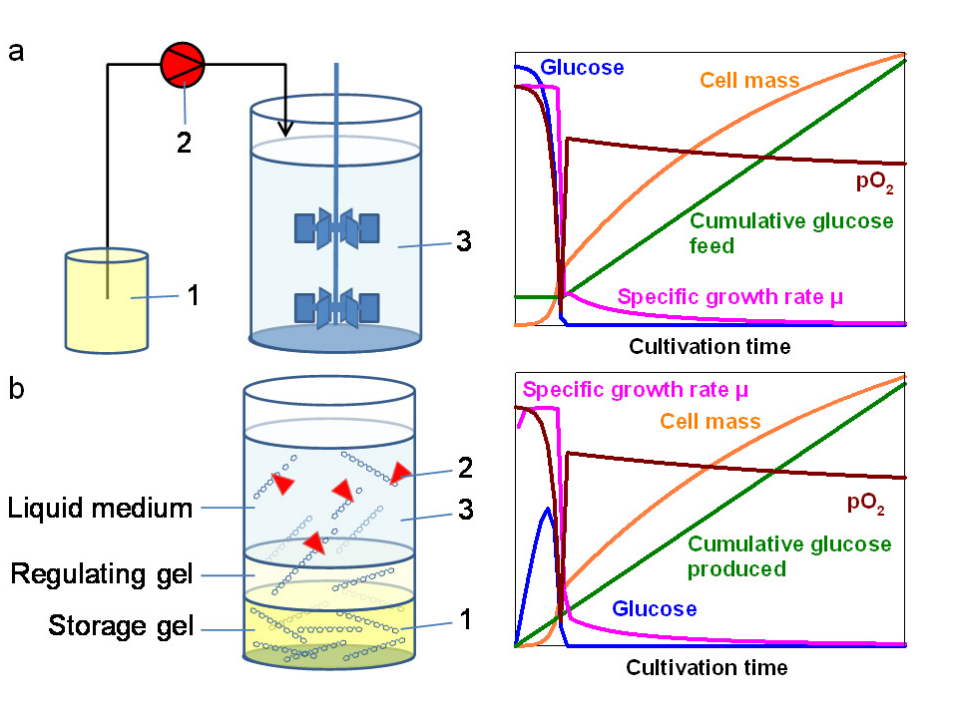
**Principle concepts of high cell density cultivation by the substrate limited fed-batch cultivation in a bioreactor (a), and by the novel enzyme controlled substrate auto-delivery system (b).** Designations: (1) substrate reservoir, (2) control system for supply of the substrate with a pump in the standard bioreactor or with a specific concentration of an enzyme in the novel auto-delivery system, (3) liquid cell culture medium. A typical fed-batch process often starts with a batch phase which is characterised by a high initial glucose concentration which steadily decreases (a). In difference, in the novel method (b) the glucose level increases in the first phase due to the glucoamylase function and low consumption by the low number of cells. In both cases (a and b) the glucose concentration is very low after the initial batch phase. The biomass increase over the time is controlled either by the pump (a), or by the enzyme (b).

In contrast to variable shaking cultures, in well controlled bioreactor scale cultivations, the fed-batch technology is mostly applied since it provides a metabolic control. In most fed-batch processes, a continuous supply of a growth limiting substrate by an external pump balances the fluxes through the cell's catabolic and synthetic pathways with the rates of energy generation (Fig. 1a, for relevant reviews see e.g. [17,18]). If glucose is used as a carbon source, the glucose consumption rate during aerobic metabolism in many organisms exceeds the capacity of the tricarbonic acid cycle and/or the regeneration of NAD^+ ^from NADH_2 _by respiration. Both of these consequences are discussed as the basis for overflow metabolism, such as acetate production in *E. coli*. The glucose consumption can be simply controlled in a bioreactor by continuous substrate feeding so that it limits the growth rate. When this principle is applied, the partial pressure of oxygen (pO_2_) in the reactor is related to the feed rate of the substrate.

Many substrate limited fed-batch processes are started as a batch process, until the initial substrate, often glucose, is consumed. In the second phase the continuous feeding of a highly concentrated glucose solution is started, in case that glucose is the limiting substrate. Different principles are used to control the substrate feed rate.

A constant specific growth rate can be ensured by an exponential feeding profile; however the pO_2 _decreases exponentially in this case. The simplest principle, a constant feed rate, is applied in most processes at least from the point onwards when the set pO_2 _limit is reached at the maximum oxygen transfer capacity of the bioreactor. The pO_2 _level is approximately constant with a constant substrate feed rate. The growth is quasi linear in this case and the specific growth rate declines steadily (see Fig. 1a).

The maximum biomass concentration in a carbon/energy substrate limited fed-batch is determined by (i) the maximum possible feed rate which can be maintained without oxygen limitation, i.e. by the oxygen transfer capacity of the bioreactor, (ii) by the effect of dilution of the bioreactor content by the feed, i.e. the substrate concentration in the feed solution should be kept as high as possible, and (iii) by the amount of glucose which is used to fulfil the needs for the cellular maintenance – that's why cultures of cells with higher maintenance only reach a lower final cell density (for discussion of maintenance effects in high cell density cultivations see [19]). With the fed-batch technology high cell densities (20 to 50 g l^-1^) can be reached at overall low specific growth rates (range between 0.05 and 0.15 h^-1^) without limitation of oxygen [20,21]. By engineering the glucose uptake system [22]*E. coli *strains capable of growing to high cell densities in batch cultures may be available in the future. Currently however the industrial high cell-density processes rely on the fed-batch principle.

In shaken cultures substrate-limited growth is not commonly applied as it is not trivial to implement a continuous well-controlled feed flow in a shaking environment. However, recently some new solutions for parallel cultures with good repeatability and growth control have been introduced into small scale (for review see [23]). The miniaturisation of bioreactors to the millilitre and sub-millilitre cultivation scale has resulted in many new exciting inventions, especially in the sensor area [24,25], but also resulted in new ways of satisfying a good oxygen transfer [26-28].

A major challenge is not only the adaptation of the fed-batch principle in the small scale, which is mostly done by intermittent feeding [29-31], but more importantly reaching high cell densities at the same time. Aside from cellular factors, the cell density in a fed-batch process is determined by the concentration of the growth limiting nutrient source in the feed. This creates a dilemma: high cell densities are only obtained when the culture is grown as glucose limited fed-batch, which needs a well controlled continuous flow of the glucose feed solution, but at the same time the feed source must be supplied in a highly concentrated form. Highly concentrated feed solutions, which contain up to 80% glucose, are used in industrial processes. In microbioreactors, the continuous feed flow and mixing of extremely small volumes of viscous solutions cannot be solved satisfactorily.

The need for continuous substrate delivery of the carbon source in aerobic cultures for high cell density cultivations has raised the interest for alternative solutions. A new innovative principle for this was first presented by Jeude et al. [32] by applying an internal substrate delivery system which works like a drug delivery system in medical applications.

The idea of supplying substrates by internal delivery systems is not new, but was already introduced by Tyrrell et al. [33] as early as 1958. They overlaid a nutrient agar phase with liquid growth medium. Later, Lübbe et al. [34] applied the concept for controlled feed of ammonia to *Streptomyces *cultures. Jeude et al. [32] packed crystalline glucose into silicon elastomer discs from which glucose is continuously released into the growth medium. Their system allows in a very easy way fed-batch cultivations in shake flasks and even in microwell plates without the need for external feeding devices. However, the glucose supply rate is not constant as the release of glucose is diffusion based. Equally important, this delivery system does not allow a simple adaptation of the glucose release rate to the oxygen transfer conditions in the culture vessel or a control of the specific growth rate.

Here we present a novel simple solution for fed-batch cultivation in millilitre and microlitre scale without the need for external feeding devices. The solution allows a simple control of the growth rate and a quasi-constant release of glucose. The novel technology combines a continuous diffusion of a metabolically inactive polymer, starch, from a gel phase into the culture medium and the enzymatic conversion of the starch to glucose substrate. In this system, the glucose release rate and the growth rate of the culture can be simply controlled by the amount of the enzyme glucoamylase in a similar way as the pump speed of the feed solution is changed to control the growth of cells in a bioreactor (Fig. 1a and 1b). In this study we demonstrate the feasibility of the method by cultivations with *Escherichia coli *and for overproduction of a heterologous protein, *Trypanosoma brucei *triosephosphate isomerase (*Tb*TIM).

## Results

### Development of the glucose auto-delivery system

The substrate auto-delivery system is based on the controlled glucose release into the liquid cultivation medium through enzymatic degradation of starch. One major objective was to reach cell densities comparable to fed-batch bioreactor cultivations. However, the amount of starch necessary to achieve this is far above the concentration which remains soluble in a liquid. Even solubilised starch easily looses it's solubility due to the interactions between the amylose chains. In addition, a high concentration of starch in the cultivation medium would decrease the oxygen transfer rate due to the increased medium viscosity. Therefore the starch concentration in the medium should be low. This practical conflict was solved by generation of a two-phase system, consisting of a gel as the starch reservoir, and a liquid cultivation medium (Fig. 1b). The gel composition was optimized so that starch diffuses just fast enough from the gel to the medium without limiting the enzyme reaction and for a high mechanical gel stability to resist vigorous shaking.

A starch concentration of 10% was embedded in the gel to provide enough glucose for a cell density of about 30 g l^-1 ^(OD_600 _≈ 100) in the liquid phase assuming that (i) all starch is available and gets processed, and (ii) one gram of glucose yields about 0.5 g cell dry weight. In initial experiments it was found that the mechanical stability of the starch gel could be increased by addition of agar (data not shown). Separate optimization of the gel for use in shake flasks and in 96-well plates resulted in different agar concentrations, 5% for shake flasks and 1.5% for 96-well plates. This gel is referred to as the storage gel.

**Figure 2:**
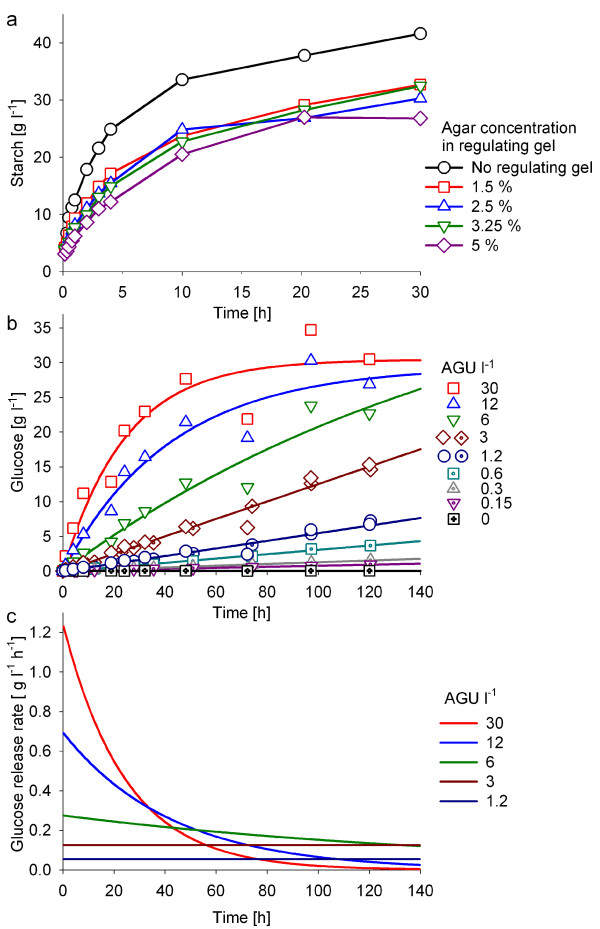
**Optimization of the novel glucose delivery system.** (a) Effect of the regulating gel and its composition on the accumulation of starch in the culture medium. Starch diffusion from the storage gel was investigated in microwell plates without addition of glucoamylase and with sterile liquid medium. The control was without regulating gel. (b) Glucose accumulation from a two-phase gel into sterile liquid medium with different amounts of glucoamylase (0.15–30 AGU l^-1^). (c) Comparison of the glucose release rates from figure (b). For the experiments the microwell plates were incubated at 37°C at a shaking speed of 750 rpm. Whole wells were harvested for each analysis.

An additional intermediate agar layer between the storage gel and the cultivation medium (referred as regulating gel) prevents the release of insoluble starch particles to the liquid medium and provides a better control of the release rate of soluble starch. The results in Figure 2a clearly show for 96-well plates that the agar concentration in the regulating gel affects the diffusion of starch into the growth medium significantly. The regulating gel demonstrated sufficient mechanical resistance and was able to sustain intensive agitation when the concentration of agar was kept above 3.25% in microwell plates and above 5% in shake flasks. The thickness of the regulating gel slightly influenced the release of starch (data not shown). Its volume was kept as small as possible to maximize the cultivation volume. A 50 μL agar layer with 1.5% agar was considered to be optimal as regulating gel for 96-well plates. A 75 ml agar layer with 5% agar was ideal for 1 litre shake flasks.

The concept of controlling the glucose release by varying the concentration of glucoamylase in the cultivation medium is demonstrated in Figure 2b for the 96-well plate system. For establishing the system we have applied a glucoamylase from *Aspergillus niger *(AG 300L) which is commercially available and characterised by good stability.

The results show a clear dependency of glucose accumulation on the glucoamylase amount in the cultivation medium over the tested range of enzyme concentrations (Fig. 2b, c). For fed-batch type cultivation experiments with *E. coli *a glucoamylase amount of 6 to 12 AGU l^-1 ^(amyloglucosidase units per liter) may be proposed as a good starting point, assuming that a release of 5 to 10 g l^-1 ^glucose over a time period of 20 hours would be related to an overall specific growth rate of 0.2 ± 0.1 h^-1^. Based on these calculations, and considering a yield coefficient of 0.5 g cell dry weight per one gram of glucose, a culture inoculated with an OD_600 _of 0.1 should growth up to an OD_600 _of 8 to 16 within 20 hours.

### Substrate auto-delivery system for fed-batch cultivation of *E. coli*

**Figure 3:**
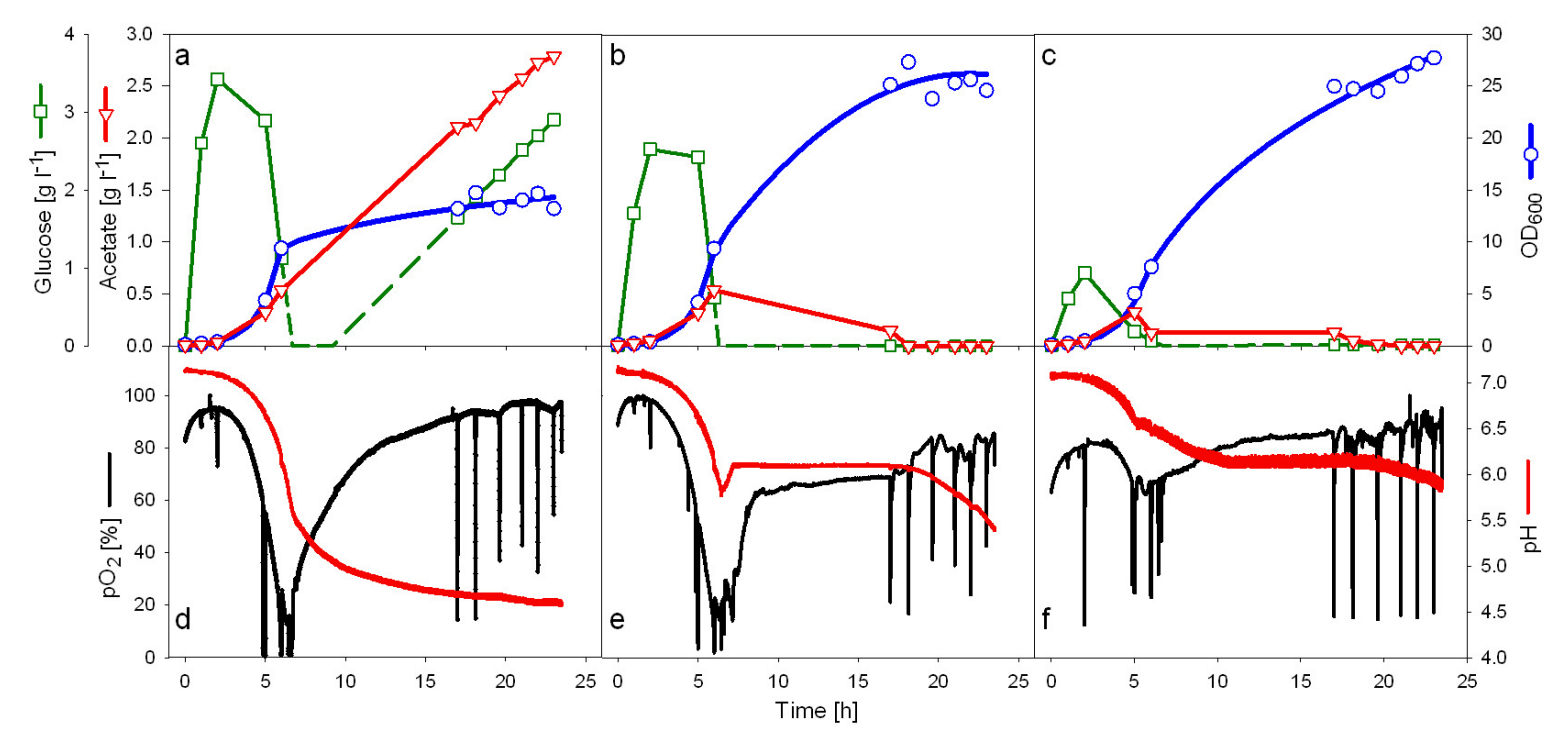
**Shake flask cultivations of *E. coli *RV308 at 37°C with the EnBase system with different amounts of glucoamylase (a, d: 60; b, e: 30; c, f: 12 AGU L^-1^).** pO_2 _and pH were analyzed in 12 sec intervals with the Senbit system. The spikes in the pO_2 _curves reflect intermediate stops of the shaker for sampling. Glucose and acetate were analysed as described in the Materials section.

After optimization of the composition of the system, the principle of the enzymatic substrate auto-delivery system was first applied in shake flask cultures. The growth behavior with differing amounts of glucoamylase was tested with *E. coli *K-12 RV308 and *E. coli *BL21(DE3) as examples. Both strains are widely used in bioproduction of recombinant proteins. The physiological events in the shake flasks were monitored by the wireless Senbit^® ^system [15] with on-line sensors for pH and pO_2_, and off-line analyses for glucose, organic acids, and the cell density (OD_600_). As expected, the cultures at all three tested glucoamylase concentrations of 60, 30, and 12 AGU l^-1 ^showed an initial batch phase, during which the glucose level was clearly not growth limiting (Figs. 3a–c). The glucose concentration first increased, and then by the higher metabolic activity at increasing cell concentrations, decreased. During the batch phase, lasting approximately for 5.5 to 7 hours, depending on the enzyme concentration, the culture density increased exponentially and the pO_2 _and pH levels decreased. The pO_2 _and pH levels decreased more in the cultures with higher glucoamylase concentration. With both glucoamylase concentrations, 30 and 60 AGU l^-1 ^(Figs. 3a, b), the cultures reached a critical pO_2 _level close to zero. Glucose limited growth after the batch phase was only established in the cultures with 12 and 30 AGU l^-1^. In the culture with the highest enzyme concentration tested glucose accumulated constantly until the end of the cultivation. In this culture, despite the availability of glucose, cell growth was inhibited and did not rise above an OD_600 _of 10. This was due to the accumulation of acetate and the connected drop of the pH value to 4.5 (Fig. 3a, d), which is non-optimal for growth of *E. coli *[35]. In contrast, the acetate level remained low and pH and pO_2 _levels were rather stable in the cultures with lower glucoamylase concentrations (12 and 30 AGU l^-1^), and also a high final cell density was obtained (OD_600 _> 25). None of these cultures showed significant accumulation of either lactate or formate, being well in agreement with the observed pO_2 _values. This indicates that the reason for the growth inhibition at the highest release rate of glucose is rather the acidification of the medium by overflow metabolism than anaerobic metabolism.

However, screening for the optimal amount of glucoamylase is easy. Without measuring glucose, acetate, or the pO_2 _in the culture, screening for highest cell density with different glucoamylase amounts will result in cultivation conditions which are still aerobic and where the acetic acid production is so low that the pH is not turned to very unfavourable conditions. As both, anaerobic metabolism [36] and overflow metabolism, can lead to a significant decrease of the biomass yield, the proper enzyme concentration can be selected straight forward by evaluation of the cell density.

**Figure 4:**
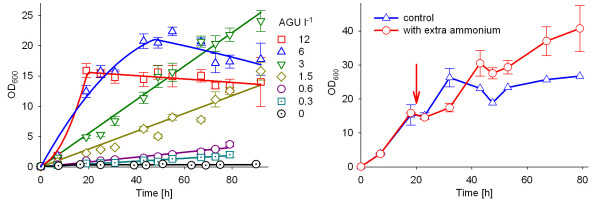
**Cultivations with EnBase of *E. coli *BL21(DE3) in 96-well plates.** (a) Effect of different amounts of glucoamylase on the growth. (b) Effect of extra addition of ammonia. Cultivations were performed at 37°C with 150 μl liquid medium per well. Different amounts of glucoamylase, as indicated in the figure, were added at the time of inoculation in (a). (b) All cultivations were performed with an initial concentration of 6 AGU l^-1 ^and extra ammonia (5 μl of a 1% stock solution) was added after 20 h of cultivation (arrow) providing a calculated extra ammonia concentration of 0.29 g ammonia per l liquid medium. The cell density (OD_600_) was analyzed from 5 μL samples in all cases. The error bars reflect cultivations in 5 parallel wells of one plate (a) and 10 parallel wells per plate (b). Evaporation per well was 0.125 μl per h^-1^.

Such screening can be readily done in 96-well plates due to the large number of possible parallel cultivations, as is demonstrated in Figure 4. In this case *E. coli *BL21(DE3) was cultivated in 150 μl liquid medium at 37°C with glucoamylase amounts ranging from 0.3 to 12 AGU l^-1 ^(Fig. 4a). As is concluded from the high cell densities and the linear growth profiles reached in the cultures with 3 to 6 AGU l^-1^, these were the highest tested enzyme concentrations which supported glucose limited cultivation over the whole cultivation period. The data show that also lower enzyme concentrations can be applied if one would aim for slow controlled growth. An enzyme concentration of 12 AGU l^-1 ^resulted in a lower cell density; probably the higher glucose release had caused either accumulation of acetic acid and a steep decrease of the pH in the culture as described above, or even stronger oxygen limitation and anaerobic metabolism.

From the relative share of nitrogen in the biomass [37] we proposed that nitrogen limits the growth at a cell density of 20 to 25 in our medium. Therefore, it was then investigated whether the cell density can be further increased by addition of additional nitrogen. Addition of ammonia ions after 20 h of cultivation, at approximately the same as the initial concentration, resulted in continued growth up to a final cell density of about OD_600 _= 40 (80 h of cultivation, Fig. 4b). The highest OD_600 _was obtained if the extra nitrogen was added as a mixture of ammonium sulphate and ammonia to correct for the decreased pH value.

These experiments document how growth can be simply optimised with EnBase cultivations for the specific shaking conditions and the biological system. The optimal amount of enzyme is selected by testing different glucoamylase concentrations and by analysing the growth profile. Most favourable growth conditions resulted in the highest cell density in the defined time window.

### Protein expression in EnBase microwell cultures at high cell densities

As there is a big interest in a parallel cultivation system for recombinant protein production (e.g. [11,38]), the novel glucose auto-delivery system was applied for that application. As an example the production of recombinant *Trypanosoma brucei *triosephosphate isomerase (*Tb*TIM) in the 96-well system was investigated. The gene had earlier been transferred into *E. coli *BL21(DE3) pET3a pLysS [39]. The test system was chosen for the reason that (i) the BL21 T7-RNA polymerase system is widely used and has even been proposed as the best consensus protocol for production of soluble recombinant proteins in *E. coli *[16], (ii) *Tb*TIM shows a high expression level in this system [39], and (iii) *Tb*TIM shows the non-desired tendency to aggregate irreversibly, which complicates the structural studies of the protein and it's variants with mutations in different amino acids [40]. We have earlier experienced that the cultivation conditions strongly affect aggregation of *Tb*TIM. Therefore it was interesting to investigate whether the growth rate at the time of induction influence the amount of product and the relative share of soluble active *Tb*TIM. A further challenge was that the optimal cultivation temperature for *Tb*TIM production is 25°C, a temperature which was not tested for the novel auto-delivery system previously, but is generally used if aggregation is a problem [16]. Aside from analysis of the cell density, samples were taken to follow the *Tb*TIM concentration by SDS-PAGE.

**Figure 5:**
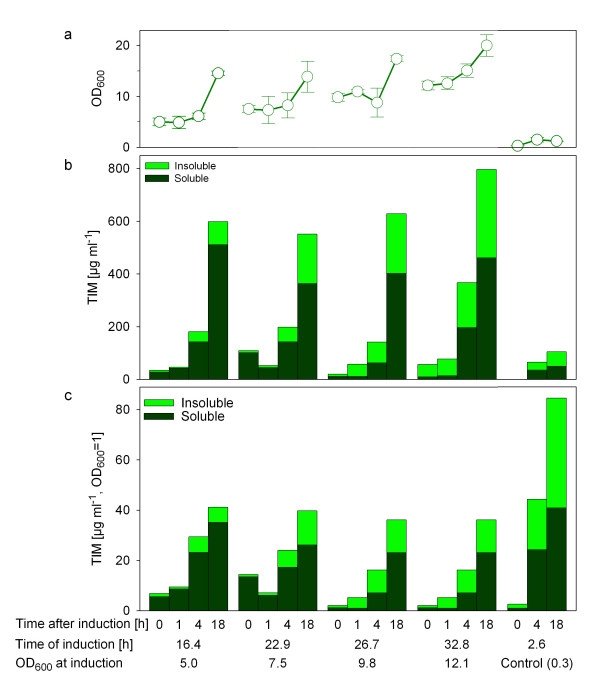
**Expression of recombinant *Tb*TIM in 96-well plates with EnBase at 25°C and induction at different cell densities.** Expression levels of *Tb*TIM are shown as a ratio of insoluble protein (light green) and the target, soluble protein (dark green). (a) Growth curves after induction; (b) Total volumetric amounts of *Tb*TIM and (c) product amounts expressed per cell unit. The protein amounts after induction are shown for four different OD_600 _levels in the novel cultivation system and for the reference culture (M9ZB, induction at OD_600 _= 0.3). Cultures were performed with 3 AGU l^-1 ^of glucoamylase. Product synthesis was induced by addition of 0.5 mM IPTG at the indicated times and cell densities, and the cultivation was continued for up to 18 hours after induction. Additional ammonia (at 26.2 h) and magnesium (at 36.85 h) were added during the cultivations as described in the Materials section.

The accumulation of soluble *Tb*TIM was increased in the EnBase cultures by a factor of 8 to 10 compared to the control on M9ZB medium without EnBase (Fig. 5, 364 to 512 μg l^-1 ^in the EnBase cultures dependent on the time of induction versus 51 μl l^-1 ^in the control culture). The production of *Tb*TIM in M9ZB medium was earlier described [41] and had given the best results so far. That is why we used it as a production standard method for different *Tb*TIM variants in shake flask cultures.

Despite the total production of *Tb*TIM per cell was lower in the EnBase cultures (possibly due to the use of mineral salt medium), the total amount of soluble *Tb*TIM was significantly higher compared to the control culture. The higher yield of total *Tb*TIM in the EnBase cultures is due to the high cell density and, unexpectedly, a higher relative share of soluble *Tb*TIM compared to the traditional batch culture system.

Although the highest amount of soluble *Tb*TIM could be harvested if the culture was induced at OD_600 _= 5.0, it is remarkable that the process is very robust in view of the time of induction (cf. Fig. 5b). The amount of harvested product deviated from the average by less than 20% for the cultures which were induced between OD_600 _5.0 (16.4 h after inoculation) and 12.1 (32.8 h after inoculation).

## Discussion

This study provides a powerful and novel method for high cell density cultivation in shaken cultures. It is based on an automatic substrate delivery principle with a simple possibility to control the substrate release.

We believe that this is the first system which, in a very simple way, transfers the advantages of the fed-batch technology into widely used cultivation vessels, including but not limited to shake flasks and microwell plates. Specifically, if compared to the delivery system from Jeude et al. [32], it extends the experimental space by giving the user the possibility to optimise the speed of glucose release, simply by changing the enzyme amount. This is important as the glucose supply rate needs to be adapted to the oxygen transfer capacity of the reactor. One may even imagine to control the glucoamylase activity during the process e.g. by changes in the pH value or addition of enzyme inhibitors, which would add an additional way of control.

In shaken systems, both oxygen transfer and pH change, are generally recognised problems and a matter of debate [16]. Cultures turn to unfavourable conditions and generally this is not even recognized. The strength of the described technique is that it does not depend on distinct monitoring tools, pumps or computer control. The optimal speed of substrate release can be easily established in a few cultivations simply by modifying the amount of added glucoamylase. It is worth mentioning that the novel auto-delivery method reflects a glucose-limited fed-batch process with a constant feed rate. More complicated feed procedures, such as exponential feeding are imaginable, but not absolutely necessary, as most glucose limited fed-batch processes include constant feed rate phases.

With the current method the cell density is limited by nitrogen limitation. This was seen in the experiments with additional supply of nitrogen (Fig. 4b) and in theoretical calculations based on the known substrate amounts in growth medium and known yield coefficients of nitrogen [37]. Eventually the extra addition of ammonia can be avoided by combining our system with the continuous ammonia supply system which was earlier developed by Lübbe et al. [34], and by this the final cell densities could be further improved. However, it should be noted that the cell densities obtained in shaken cultures may not reach the high levels of well aerated bioreactors (100 to 300 g l^-1 ^cell dry weight, [21]) due to the significantly lower oxygen transfer coefficient in shaken cultures.

We are aware that the application is neither limited to *E. coli *nor to the use of starch/glucoamylase, but the principle idea opens a wide field of possible applications and enzyme-substrate combinations. If EnBase is applied for organisms which produce own amylases, such as *Bacillus subtilis*, it may be more difficult to control their growth. However, initial results (not shown, separate publication considered) suggest that high cell densities can be obtained also with *Bacillus subtilis*.

The potential of EnBase was clearly demonstrated with the example of recombinant *Tb*TIM. Surprisingly, by applying the glucose limited fed-batch principle not only the amount of product was increased by the higher cell density, but also the relative amount of soluble to insoluble *Tb*TIM was significantly higher, with a decreasing tendency when the culture was induced at higher density.

## Conclusion

The EnBase method is robust and simple-to-apply for high cell density cultivation. The enzymatically controlled release of the growth substrate provides the possibility to control the growth rate. The application of a glucose-limited fed-batch technique in shaken cultures solves problems with uncontrolled oxygen limitation and severe pH drop. In parallel it provides the basis for enhanced cell densities. EnBase has been shown to work in 96-well plates and shake flasks, and we believe that it easily can be adapted to different cultivation formats. Therefore it should be a useful tool especially in high throughput applications.

Aside from the optimization of recombinant processes with the chance for direct scalability, there is an equally large potential for applying EnBase in the parallel production of different proteins in multiwell plates. Since significantly higher cell densities are obtained compared to traditional batch cultures, EnBase has a large potential for partially replacing labour-intensive shake flasks by multiwell plates e.g. for high throughput crystallization or screening of enzyme libraries. Enabling the scale-down of cultures from shake flasks to deepwell plates by the increased cell density, the method may be also beneficial in plasmid production, e.g. for genome sequencing where shake flask cultures are the state of the art.

## Methods

### Strains and plasmids

*E. coli *RV308 (β*lacX*74 *gal*ISII::OP308 *strA*, ATCC 31608), *E. coli *BL21(DE3), and the plasmid containing strain *E. coli *BL21(DE3) pLysS pET3a*Tb*TIM [41] were used in this study.

### Cultivation media

All cultivations with the novel substrate delivery system were performed in glucose-free mineral salt medium (MSM, [42]) with the following composition (per litre): 2.0 g Na_2_SO_4_, 2.7 g (NH_4_)_2_SO_4_, 0.5 g NH_4_Cl, 14.6 g K_2_HPO_4_, 3.6 g NaH_2_PO_4 _× H_2_O, 1.0 g (NH_4_)_2_-H-citrate, 2 mM MgSO_4_, 0.1 g l^-1 ^of thiamine hydrochloride, and 2 ml l^-1 ^trace element solution [43]. Additionally for precultures this medium contained 5 g l^-1 ^glucose (glucose-MSM). M9ZB medium contained (per litre): 10 g casein hydrolysate (Sigma-Aldrich, St. Louis, USA), 5.0 g NaCl, 1.0 g NH_4_Cl, 3.0 g KH_2_PO_4_, 3.2 g Na_2_HPO_4_, 4 g glucose, and 1 mM MgSO_4_. 100 μg ml^-1^ampicillin and 35 μg ml^-1 ^chloramphenicol were added in order to maintain selective pressure for the pET3a*Tb*TIM and pLysS plasmids, respectively.

### Gel system

The substrate auto-delivery system was based on a two-phase gel, the storage gel contained soluble potato starch (Sigma-Aldrich, St. Louis, USA) and agar (BD, Bacto Agar, Franklin Lakes, USA) as a stabilizing agent, the regulating gel only contained agar but no starch. In microwell plates (Perkin Elmer Spectra Plate™ – TC 96, Waltham, USA) the storage gel contained 10% starch and 1.5% agar. The gel was prepared by suspending the starch in a small volume of distilled water at room temperature. The suspension was poured into a larger volume of intensively mixed hot distilled water (≈95°C). After mixing the hot suspension for 20 to 30 min intensively by keeping it at about 90°C, the agar was added directly to the hot starch suspension, which was then filled with distilled water to the final volume. The upper regulating gel was prepared by resuspending the agar, 1.5 to 5% for microwell plates and 5% for shake flasks, in distilled water. The gels were autoclaved separately for 20 min at 121°C and afterwards stored at room temperature. The microwell plates were filled under aseptic conditions, with 100 μl of warm liquefied storage gel. After solidification 50 μl of warm regulating gel was added. The 1 l shake flasks contained 100 ml storage gels and 75 ml regulating gel layers.

### Cultivation conditions

Precultures were prepared from frozen glycerol-stocks in 100 ml Erlenmeyer flasks containing 10 ml of glucose-MSM by overnight cultivation of the respective strains at 37°C on an orbital shaker (180 rpm). For inoculation the preculture medium was removed by centrifugation (23°C, 3200 × g, 20 min) and the pellet was resuspended in glucose-free mineral salt medium. The preculture medium of *E. coli *BL21(DE3) pLysS pET3a*Tb*TIM was removed by centrifugation (23°C, 3200 × g, 4 min) and the pellet was suspended either in glucose-free MSM or M9ZB.

Shake flask cultures with the glucose auto-delivery system were performed in 1 litre Erlenmeyer flasks with 4 baffles (Glasgerätebau Ochs, Bovenden-Lengelern, Germany) with side necks for the placement of the pH and oxygen sensors (cf. [15]) containing the two-phase gel and a total of 165 ml of glucose-free mineral salt medium. The indicated amounts of *Aspergillus niger *glucoamylase (Amylase AG 300L, Novozymes, Bagsværd, Denmark) were added after inocculation and the culture was immediately started (37°C, 180 rpm).

For cultivation in 96-well plates (Perkin Elmer Spectra Plate™ – 96 TC, Waltham, USA) the outer surrounding wells were not used for cultures but filled with water to limit evaporation. For cultivation the inner, gel-containing wells were inoculated with 150 μl of glucose-free MSM containing the cells and glucoamylase in the respective concentrations. The cultivations were performed with a Variomag Thermoshaker (with TEC-controller 485, Inheco, Munich, Germany) at 750 rpm. To minimise evaporation the plate was placed in a humid cabinet controlled at approximately 80% humidity. Under these conditions the evaporation was about 1 μl per well and hour at 37°C.

The cultivations of *E. coli *RV308 and *E. coli *BL21(DE3) were performed at 37°C and the cultivation of *E. coli *BL21(DE3) pLysS pET3a*Tb*TIM was performed at 25°C. Liquid culture controls were placed into the same plate with 150 μl M9ZB but without gels. Product synthesis was induced by addition of 0.5 mM IPTG. Additional nitrogen (at time point 26.2 h) and magnesium (at time point 36.85 h) were added to the cultivation of *E. coli *BL21(DE3) pLysS pET3a*Tb*TIM by adding NH_3 _(as 25% solution) and (NH_4_)_2_SO_4_, and MgSO_4 _(as 0.5 M solution) to final concentrations at 0.033%, 2.6 g l^-1^, and 3.3 mM, respectively.

### Analytical procedures

For medium analysis cells were removed by centrifugation in a microfuge (16 000 × g, 3 min, 4°C) and the clear supernatants were used for analysis.

The starch concentration was analysed by the amount of glycosyl residues after complete acid hydrolysis and following glucose analysis, according to the following procedure (modified from Ritte et al. [44]): 100 μl of sample was hydrolyzed by addition of an equal volume of 2 N HCl solution at 100°C for 2 hours. The solution was then neutralized with 100 μl of 2 N NaOH and 100 μl of 0.5 M potassium phosphate buffer.

Glucose was analysed with a YSI 2700 Select Biochemical Analyzer (YSI Inc., Yellow Springs, USA).

Acetate was analysed by high-performance liquid chromatography (HPLC). Medium samples were stored at -20°C until HPLC analysis. Samples were thawn on ice, incubated at 80°C for 5 min to solubilise possible precipitates, centrifuged at 16 000 × g for 5 min at +4°C, and finally filtered with 0.2 μm cellulose filters. The HPLC analysis was done with a Merck-Hitachi HPLC-system (Model D-6000) and ICSep COREGEL 87H3 organic acids column (Transgenomic Inc., Omaha, U.S.A.). Running buffer was 0.01 N H_2_SO_4_. Detection of organic acids was performed with a UV-VIS detector (L-4250, Merck Hitachi) at 210 nm. Data analysis was performed by the D-7000 HPLC System Manager (version 3.1.1, Hitachi) software.

Cell growth was followed with a spectrophotometer after the dilution of culture samples in growth medium at either 600 nm (OD_600_) for samples from shake flask cultures with an UltroSpec Pro 2100 UV/Visible Spectrophotometer (GE Healthcare, Buckinghamshire, UK), or at 490 nm (OD_490_) from the 96-well plate cultures with a Victor^3 ^Multiwell Plate Reader (Perkin Elmer, Waltham, USA) and a sample volume of 5 μl. 1 unit of OD_490 _corresponds to 0.73 of OD_600_. All cell density values are presented as OD_600 _values. One unit of OD_600 _corresponds to a dry cell weight of 0.3 g l^-1 ^[36].

The SENBIT^® ^wireless system (teleBITcom GmbH, Teltow, Germany) was used in the shake flask cultures to follow the pO_2 _and pH continuously in 12 s intervals as described earlier [15].

### Quantification of *Tb*TIM

Overexpression and purification of *Tb*TIM, to provide a standard of pure protein for the SDS-PAGE analysis, was done as previously described [41]. For samples the culture media was removed by centrifugation (16 000 × g, 3 min, 4°C) and the cell pellets were frozen at -20°C. For analysis the cells were disrupted simply by the action of the internal T7-lysozyme by suspending them in 50 μL lysis buffer (20 mM Tris, 100 mM NaCl, pH 8) with addition of MgCl_2_, DNAse and RNAse as described earlier [41]. Insoluble cell debris (IN) was separated from the soluble cell debris (SP) by centrifugation at 16 000 × g (30 min). The amounts of cellular debris suspensions were normalized by dilution with the mentioned buffer, without the additives, on the basis of lowest optical density at OD_600_. The IN was washed twice with the same buffer by re-suspending and centrifugation at 16 000 × g (15 min). The IN was finally re-suspended in 50 μl of 6 M urea. Both samples, IN and SP, were applied to SDS-PAGE. As a reference pure *Tb*TIM (dissolved in the same buffer for the SP samples and dissolved in 6 M urea for the IN samples) was applied in different concentration on the same SDS-PAGE gel. The bands were visualized by coomassie brilliant blue staining and by scanning with Image scanner III and band intensities were quantified with the ImageQuant 5.2 software (both GE Healthcare, Buckinghamshire, UK). Known *Tb*TIM quantities were used to calculate the amount of *Tb*TIM on the sample gels (SP and IN).

## Competing interests

The authors declare that they have no competing interests.

## Authors' contributions

JPP carried out most of the experiments and drafted the manuscript. RW participated in the experiments with the 96-well plates. MC performed the protein analysis. JS participated in the drafting of the manuscript. AV and PN conceived of the study, and participated in its design, coordination, and drafting of the manuscript. All authors read and approved the final manuscript.
